# FindAdapt: A python package for fast and accurate adapter detection in small RNA sequencing

**DOI:** 10.1371/journal.pcbi.1011786

**Published:** 2024-01-22

**Authors:** Hua-Chang Chen, Jing Wang, Yu Shyr, Qi Liu

**Affiliations:** 1 Department of Biostatistics, Vanderbilt University Medical Center, Nashville, Tennessee, United States of America; 2 Center for Quantitative Sciences, Vanderbilt University Medical Center, Nashville, Tennessee, United States of America; University of Wisconsin, Madison, UNITED STATES

## Abstract

Adapter trimming is an essential step for analyzing small RNA sequencing data, where reads are generally longer than target RNAs ranging from 18 to 30 bp. Most adapter trimming tools require adapter information as input. However, adapter information is hard to access, specified incorrectly, or not provided with publicly available datasets, hampering their reproducibility and reusability. Manual identification of adapter patterns from raw reads is labor-intensive and error-prone. Moreover, the use of randomized adapters to reduce ligation biases during library preparation makes adapter detection even more challenging. Here, we present FindAdapt, a Python package for fast and accurate detection of adapter patterns without relying on prior information. We demonstrated that FindAdapt was far superior to existing approaches. It identified adapters successfully in 180 simulation datasets with diverse read structures and 3,184 real datasets covering a variety of commercial and customized small RNA library preparation kits. FindAdapt is stand-alone software that can be easily integrated into small RNA sequencing analysis pipelines.

## Introduction

Small RNA sequencing (small RNA-seq) is commonly used to profile the full spectrum of small RNAs, including microRNA (miRNA), Piwi-interacting RNA (piRNA), small interfering RNA (siRNA), tRNA, rRNA, and small nuclear RNA (snRNA) [1–7]. Small RNAs, typically 18 to 30 bp long, are generally shorter than sequencing reads, resulting in adapters included in raw reads. Therefore, the first essential step for small RNA-seq analysis is adapter trimming and recovery of true sequences [1,8,9].

There are many tools developed to trim adapters that generally require adapter information as input, such as Cutadapt [10], FASTX [11], AdapterRemoval [12], and Trimmomatic [13]. However, adapter information is not mandatory when data are deposited into public databases like the NCBI Gene Expression Omnibus (GEO). A recent study reported that around 53% of NCBI Sequence Read Archive (SRA) entries lack adapter-related information [14]. Even when adapter sequences are provided, it is often difficult to extract them from the record automatically and streamline the analysis. Manually retrieving adapter sequences from literature or raw reads is labor-intensive and error-prone, especially when there are many datasets to be analyzed. The absence of readily available adapter information greatly hampers the reproducibility and reusability of publicly available datasets.

**Fig 1:**
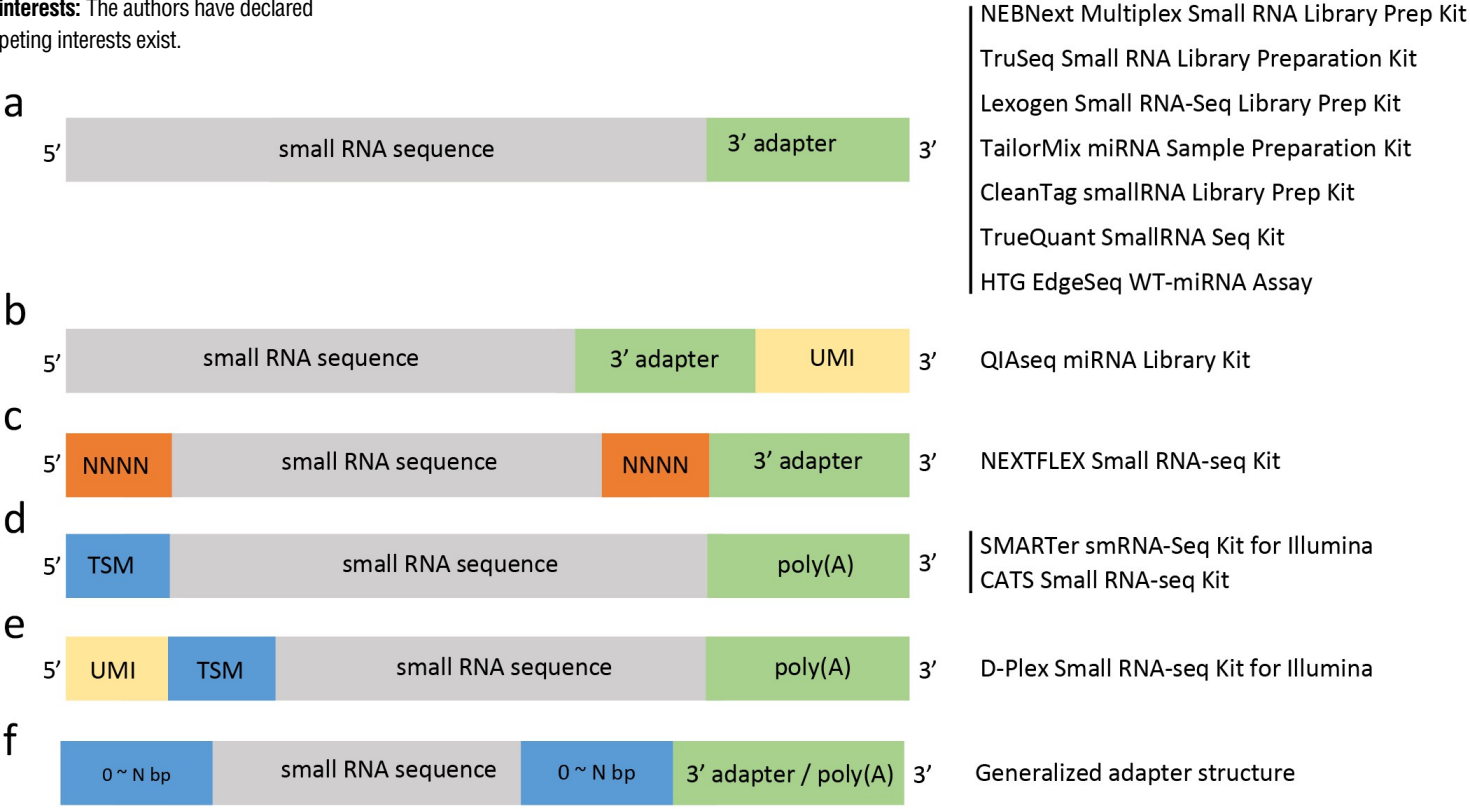
Read structures from different small RNA library preparation kits. (a) The 3’ adapter right after the true small RNA sequence by seven Kits. (b) The true small RNA sequence followed by the 3’ adapter and the UMI of 12bp by the QIAseq Kit. (c) The true small RNAs sequence surrounded by random tetramer (NNNN) at both 5’ and 3’ ends, and the 3’ adapter sequence after the 3’ random tetramer by the NEXTFLEX Kit. (d) The true small RNA sequence surrounded by poly(A) tail at the 3’ end and a TSM (template switching motif) at the 5’ end for the SMARTer and CATS Kits. e) The UMI sequence of 12bp at the 5’ before the TSM by the D-Plex Kit. f) The generalized read structure from all kits.

Different small RNA-seq library preparation methods create different adapter patterns. Fig 1 illustrates five adapter patterns and read structures generated from 12 small RNA library preparation kits. Among them, two strategies are used to mitigate sequence-specific biases during the ligation step [1,15–20]. One is to introduce random sequence at both 5’ and 3’ end, such as NEXTFLEX Small RNA-seq Kit with random tetramer (Fig 1c). The other is template switching, which contain non-template oligo sequence at the 5’ end and poly(A) tail at the 3’ end, such as SMARTer, CATS and D-Plex Small RNA-seq Kits (Fig 1d and 1e). Diverse read structures, randomized adapters, and low complexity regions make adapter detection challenging.

Several tools have been developed to identify adapter sequences from raw reads. For examples, Atropos [21], fastp [22], DNApi [23], and EARRINGS [24] count k-mers in reads to infer adapter sequences. However, k-mer counting methods do not consider random-mers at each side of the ligation site, such as the random tetramer at both the 5’ and 3’ ends produced by the NEXTFLEX Kit (Fig 1c). Additionally, most methods struggle to identify adapters with low complexity, such as the poly(A) tail introduced when libraries are prepped with SMARTer or CATS Kit (Fig 1d). The latest approach, adapt_find, uses a BLAST-based [25] strategy to identify adapter sequences [26]. It blasts reads against the reference genome to locate small RNA sequences and then infers the adapter pattern, which is computationally intensive and time-consuming. Although it is supposed to work theoretically, the package failed to return any results due to one logic error on the BLAST E-value in the script.

Here, we developed FindAdapt, a fast and light-weight Python package that identifies complex and various adapter patterns accurately without prior information. FindAdapt is far superior to existing tools, with 100% accuracy in simulated and real datasets. FindAdapt makes it easy to streamline small RNA-seq analysis and provides a hassle-free way to analyze a large number of datasets, even those generated by different commercial and customized preparation kits.

## Design and implementation

FindAdapt is designed to identify any adapter patterns, including but not limited to the variety of read structures generated by different small RNA-seq preparation kits. Fig 1 illustrates read structures from 12 commonly used kits. The simplest structures are generated by NEBNext, TruSeq, Lexogen, TailorMix, CleanTag, TrueQuant, and HTG EdgeSeq WT-miRNA, wherein the 3’ adapter immediately follows the true small RNA sequence (Fig 1a). The QIAseq miRNA library kit introduces a UMI sequence after the true small RNA sequence and the 3’ adapter (Fig 1b). NEXTFLEX adds random tetramer at both 5’ and 3’ sides of the true small RNA sequence to greatly reduce sequence biases, followed by the 3’ adapter sequence (Fig 1c). The template switching based kits, such as SMARTer, CATS, and D-plex, have the poly(A) at the 3’ end, and the template switching motif (TSM) at the 5’ end of the true small RNA sequence (Fig 1d and 1e). The template switching motif is generally a random sequence of 3 bp. The D-plex kit also adds the UMI sequence of 12 bp before the TMS (Fig 1e). The generalized structure of various kits is illustrated in Fig 1f, which includes 0-N bp random-mers at the 5’ end, 0-N bp random-mers at the 3’ end and followed by the 3’ adapter sequence or poly(A).

**Fig 2:**
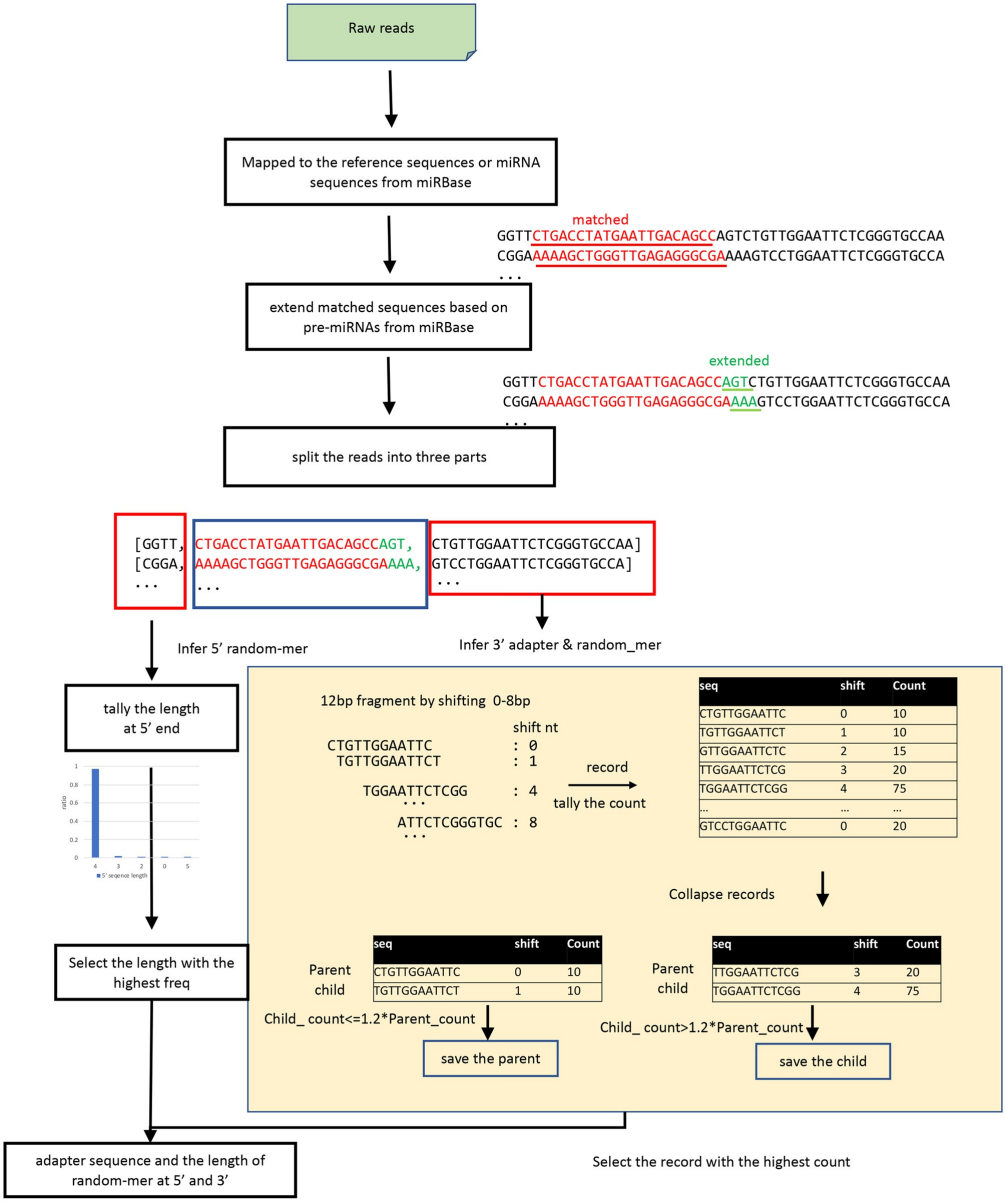
An overview of FindAdapt workflow.

The workflow of FindAdapt is shown in Fig 2. To identify adapters without prior information about library preparation, the essential step is to locate the true small RNA sequence. Rather than BLAST against the reference genome to find true sequences, FindAdapt locates true miRNAs in raw reads using the Aho-Corasick algorithm [27], which is more computationally efficient and faster. The Aho-Corasick algorithm builds a fast finite state machine of all the given reference sequences for executing searches in linear time, which is one of the most powerful algorithms for searching for patterns in a large set of sequences. The reference sequences are provided by users via the ‘-seq’ option or derived from miRBase v22.1 automatically from the organism specified by users. After locating regions of small RNAs, FindAdapt extends mapped regions based on pre-miRNA sequences from miRBase and then uses the mapped and extended regions to further define the exact boundary between true reads and adapters. FindAdapt splits reads into three regions: the upstream, the mapped and extended region, and the downstream. The upstream sequence is used to infer the 5’ random-mer length, while the downstream sequence is used to infer the adapter sequence and 3’ random-mer length. Each downstream sequence is shifted by 0-m bp (default m = 8) from the 5’ end to generate a n-bp fragment (default n = 12). The default value of n set to 12 is because the adapter sequence is either longer than 12bp or empty, and 12bp is long enough to exclude random matches. FindAdapt records each n-bp fragment along with its shifted position and tallies counts from all downstream sequences (Fig 2). FindAdapt then collapses records based on their relationships to find the most likely adapter pattern. The record with (k+1)-bp shift is the child of a record with k-bp shift if its sequence can be generated from the sequence of that record by shifting one bp. For example, the record TGTTGGAATTCT with 1-bp shift is the child of the record CTGTTGGAATTC with 0-bp shift. To increase the speed, FindAdapt first chooses the records with the top 5 highest counts in each shifted position. FindAdapt then collapses those top5 records by constructing their parent-child relationships. FindAdapt saves the child record to the candidate pool if the ratio between the count of the child and the parent is greater than a certain cutoff (default: 1.2); otherwise, the parent record is stored. The default cutoff of 1.2 is based on the fact that sequencing errors may be over 10% but less than 20%. FindAdapt finally reports the record with the highest count, where the 12bp fragment is the adapter sequence and its shifted position is the length of the 3’ random-mer (Fig 2) (Algorithm 1 in S1 Text). To be noted, FindAdapt only scans the first 10K reads and stops if it successfully obtains 1,000 matches to the reference; otherwise, it keeps loading 10K reads until it finds enough number of matches.

FindAdapt includes miRBase v22.1 in the package, from which it automatically derives miRNA sequences to use as the reference. Therefore, it supports all 271 organisms that miRBase contains. FindAdapt is implemented in Python 3 (> = 3.6) with multi-threaded support. No installation or dependencies are required. FindAdapt can identify adapters for one dataset or multiple datasets simultaneously. Its input is either a single FASTQ file or a tab-delimited file list, in which the first column is the study name or ID and the second column is the path to the FASTQ files. The output is the length of the random-mer at the 5’ and 3’ ends and the adapter sequence. In addition, FindAdapt provides the option to run adapter detection and trimming in one step. FindAdapt uses Cutadapt as the default trimming software [10], which is executable if its installation directory is listed in the $PATH environment variable or provided by the -pw_cutadapt argument.

## Results

### Performance on simulation datasets

We simulated various read structures to evaluate the performance of FindAdapt. We generated nine scenarios: 1) a simple read structure with only the 3’ adapter sequence; 2) 1~5bp random-mers at 5’ end and the 3’ adapter sequence; 3) 1~5bp random-mers at 3’ end and the 3’ adapter sequence; 4) 1~5bp random-mers at both 5’ and 3’ end and the 3’ adapter sequence; 5) The poly(A) tail; 6) 1~5bp random-mers at 5’ end and the poly(A) tail; 7) 1~5bp random-mers at 3’ end and the poly(A) tail; 8) 1~5bp random-mers at both 5’ and 3’ end and the poly(A) tail; 9) no adapters. Scenarios 1–8 included, but were not limited to, adapter patterns generated by commonly used library preparation kits. Scenario 9 served as a negative control in that adapters were not included in the reads. The simulated datasets were created by randomly sampling 10,000 post-trimmed reads from a real small RNA-seq dataset (SRA ID: SRR6502962), which mimicked realistic levels of sequencing errors. To build the random-mer sequence at the 5’ and/or 3’ ends, a number ranging from 1–5 was randomly chosen to determine its length (n), and then [A, T, C, G] was randomly sampled n times to generate the random-mer sequence. For scenarios 1–4, the 3’ adapter sequence was randomly chosen from an adapter pool compiled from real datasets with 0.25% sequencing error [28]. For scenarios 5–8, 12-nt poly(A) was used as the 3’ adapter. Random nucleotides were added to the tail to reach 50 bp if simulated reads were shorter than 50 bp, which is the read length of the real small RNA-seq dataset.

We generated 20 datasets for each scenario. The approach is successful if it recognizes both the length of the random-mer and the 3’ adapter sequence correctly when there are adapters (scenarios 1–8). For negative controls, as in scenario 9, the approach is successful if it reports random-mers of length zero and empty 3’ adapter sequences, or if it doesn’t return any adapters.

**Fig 3:**
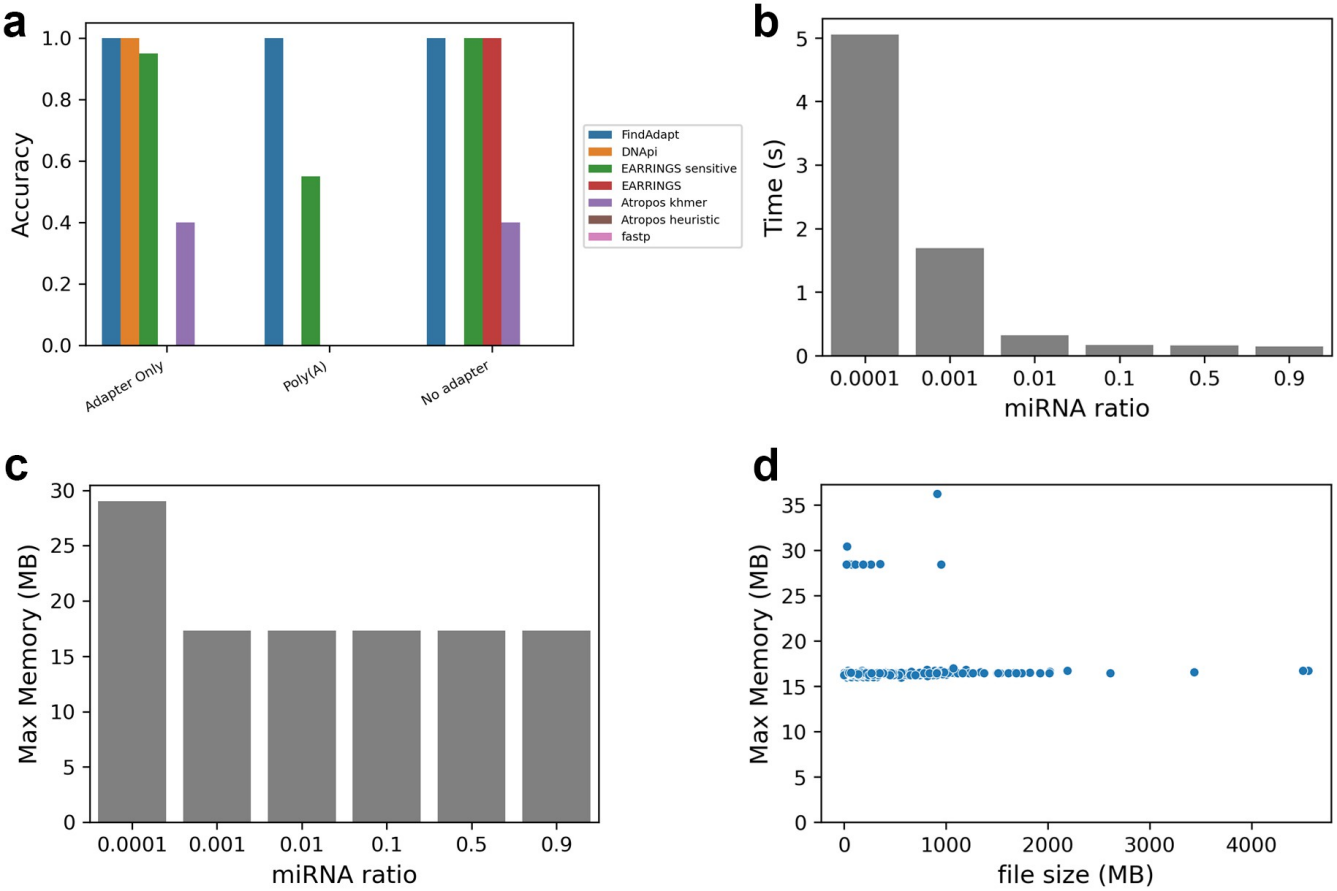
The performance of FindAdapt on simulation datasets. (a) The accuracy of FindAdapt, DNApi, EARRINGS_sensitive, EARRINGS_default, Atropos_khmer, Atropos_heuristic, and fastp on scenarios 1 (adapter only), 5 (poly (A) tail), and 9 (no adapter) where random-mers were not inserted. (b) The computational time of FindAdapt when miRNA percentages range from 0.0001 to 0.9. (c) The memory usage of FindAdapt when miRNA percentage range from 0.0001 to 0.9. (d) The memory usage of FindAdapt when file sizes range from 10Mb to 3,000Mb.

Notably, FindAdapt achieved 100% accuracy for all scenarios involving 180 datasets. We then compared the performance of FindAdapt with DANpi, EARRINGS_sensitive, EARRINGS_default, Atropos_khmer (default), Atropos_heuristic, and fastp only on scenarios 1, 5 and 9 since they cannot handle random-mers at the 5’ and/or 3’ ends (Fig 3a). EARRINGS_sensitive and Atropos_heuristic are more sensitive but computationally intensive than their default modes. In contrast, Atropos_heuristic and fastp failed in every scenario, even in the simplest scenario 1, where the regular 3’ adapter immediately followed the true sequence. DNApi achieved 100% accuracy in scenario 1, but failed in scenario 5 with poly(A) tail as adapters and scenario 9 by reporting false adapters when none were included. EARRINGS_sensitive achieved 95% accuracy in scenario 1 and 55% accuracy in scenario 5 with poly(A) tail. It successfully reported no adapters in scenario 9. EARRINGS_default worked by not returning adapters in scenario 9, but failed in scenarios 1 and 5. Atropos_khmer only achieved 40% accuracy in scenarios 1 and 9 and failed in scenario 5 (Fig 3a).

In summary, FindAdapt is far superior to existing approaches (Fig 3a). FindAdapt successfully identified adapters with random-mers at the 5’ and/or 3’ ends, which were not considered in existing approaches. For scenarios without random-mers at the 5’ and 3’ ends, only EARRINGS_sensitive identified poly(A) tails with low accuracy (55%) at the cost of much longer computational time. It took FindAdapt 0.2s to analyze one typical dataset in scenario 5, compared to 7.6s for EARRINGS_sensitive (>35 times). Additionally, Atropos_heuristic, Atropos_khmer, DNApi, and fastp reported false adapters when no adapters existed (scenario 9 in Fig 3a).

We further evaluated FindAdapt in terms of computational time and memory usage. We simulated small RNA-seq datasets with different miRNA percentages and file sizes. The simulated datasets were generated from a real dataset by randomly sampling miRNA reads and non-miRNA reads. FindAdapt was very fast, only taking less than 1s to process most datasets. Additionally, we observed that the computational time of FindAdapt was uncorrelated to the file size but negatively correlated to miRNA percentages in the data (Fig 3b). More sequences were required for screening to find 1,000 matches if a lower percentage of miRNAs was present. In the simulated datasets, for example, it took 0.17s for FindAdapt to analyze a dataset with 10% miRNA, whereas it took ~5s to analyze a dataset with 0.01% miRNA (Fig 3b). FindAdapt requires very little memory to run, and its memory usage is invariant with respect to the miRNA percentage and file size unless the miRNA percentage is extremely low (< = 0.01%) (Fig 3c and 3d). In summary, FindAdapt is fast and memory-efficient.

### Performance on real datasets

**Fig 4:**
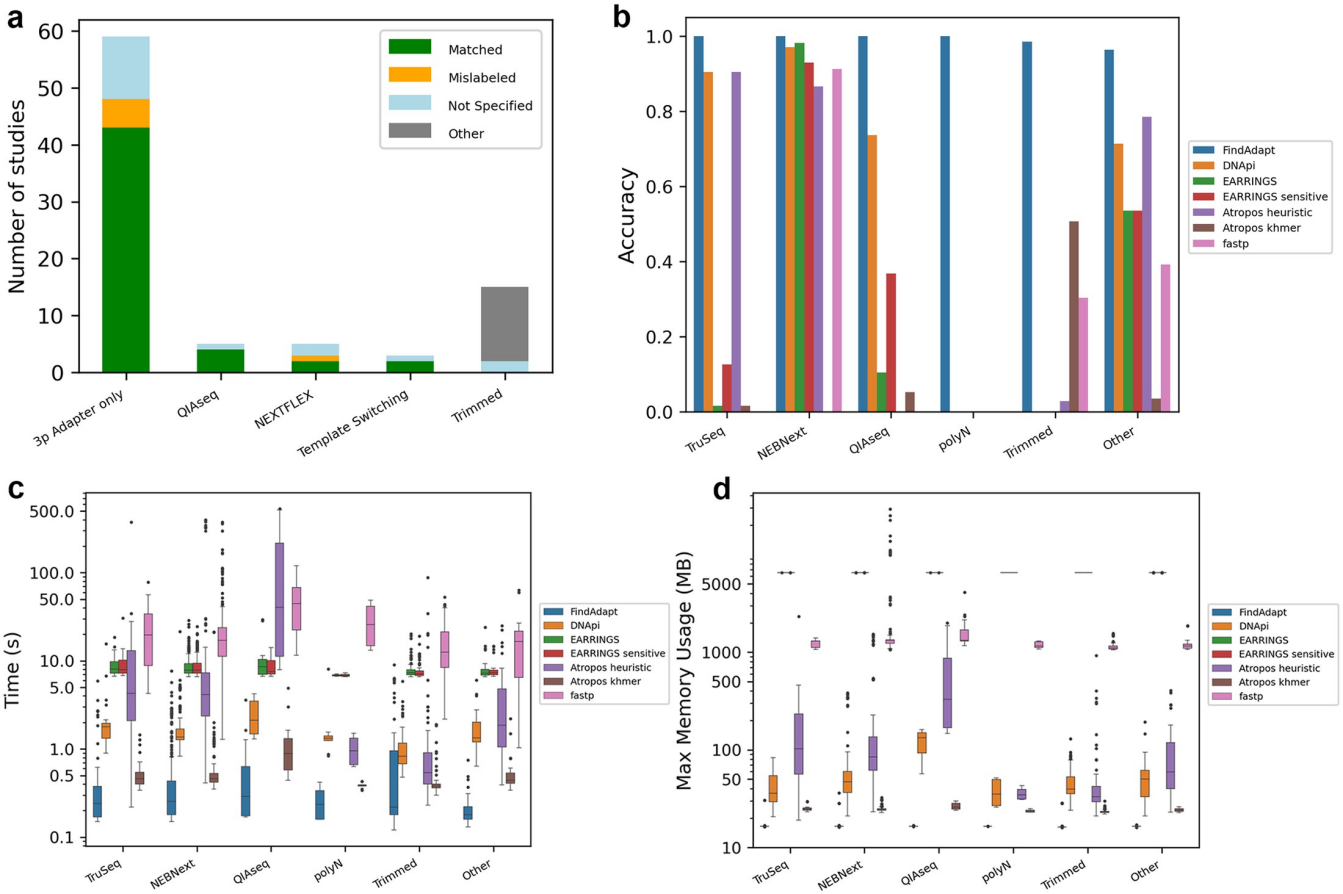
The performance of FindAdapt on the 87 human small RNA-seq studies. (a) The comparison of adapters identified by FindAdapt with the information provided in original studies in each type of adapter patterns. Matched (denoted in green) means the adapter identified by FindAdapt match the kit information provided in the original study. Mislabeled (denoted in orange) means the kit information provided by the original study was incorrect but FindAdapt identified adapters correctly. Not specified (denoted in blue) indicates the kit information was not provided in the original study. Other (denoted in gray) suggests that the kit information was provided but the reads were already post-trimmed. (b) The accuracy of FindAdapt and other methods on the 77 studies where read structures did not contain random-mers at the 5’ and/or 3’ ends. (c) The runtime of FindAdapt and other methods; d) The memory usage of FindAdapt and other methods.

We applied FindAdapt to 87 GEO [29] datasets involving 3,148 samples from extracellular vesicle-centered studies, which are challenging to analyze due to low percentages of miRNAs and high contamination. They covered a variety of adapter patterns and read structures (Fig 4a), which reflect a realistic situation when researchers analyze public datasets (S1 Table). Of these datasets, 70 studies provided information on adapters or small RNA library preparation kits, including widely used kits (NEBNext, TruSeq, QIAseq, and SMARTer smRNA-seq Kit) as well as rarely used ones (ion total RNA-seq Kit v2, TrueQuant SmallRNA Seq Kit, and HTG EdgeSeq WT-miRNA Assay) and customized adapters (GSE158948 and GSE72183) [30,31]. 17 studies did not specify the adapter or kit information (Fig 4a). For the 70 studies with kits or adapters available, FindAdapt identified patterns matching the provided information in 51 datasets (including two studies with customized adapters), mismatched in six datasets, and reported no adapters in 13 datasets (S1 Table). Manually checking raw reads, we found that incorrect adapters or kits were provided in the six studies and post-trimmed reads were deposited in the 13 studies. For the 17 studies lacking information, we generated miRNA count tables very close to those provided in the GEO, demonstrating that the identified adapters were correct (Fig 4a and S1 Table). In summary, FindAdapt identified adapters in all 87 studies accurately (Fig 4a).

We compared the performance of FindAdapt with DANpi, EARRINGS_sensitive, EARRINGS_default, Atropos_khmer, Atropos_heuristic, and fastp on the 77 studies where random-mers were not included in the 5’ and/or 3’ ends. Consistent with simulation results, FindAdapt is far superior to other approaches (Fig 4b). Although DNApi obtained good performance on datasets prepared by TruSeq, NEBNext, QIAseq, and other kits, it failed when adapters were already trimmed or contained poly(N). EARRINGS and EARRINGS_sensitive performed poorly on most studies except those from the NEBNext kit. This was mainly due to the fact that the two approaches reported the adapter from the NEBNext kit as the default when no adapters were detected. Atropos_heuristic performed well on studies produced by TruSeq, NEBNext, and other kits. However, it failed to identify adapters prepared by QIAseq and poly(N) tail, and it reported adapters falsely when no adapters were included. Atropos_khmer failed in most studies, and fastp only worked well on specific adapters generated by the NEBNext kit (Fig 4b).

In terms of computational time and memory, FindAdapt is much faster and more memory-efficient than other approaches (Fig 4c and 4d). It took FindAdapt 0.1s–9s (median = 0.2s) to process every dataset, followed by Atrophos_khmer 0.3s–21.4s (median = 0.4s), DNApi 0.5s–22s (median = 1.4s), Atrophos_heuristic 0.2s–530.5s (median = 3.2s), EARRINGS (default and sensitive) 6.6s–30.7s (median = 7.6s), and fastp 1s–276s (median = 17.1s) (Fig 4c). FindAdapt required memory of 16–36 Mb (median = 16 Mb), followed by Atrophos_khmer 22–32 Mb (median = 24 Mb), DNApi 20–385 Mb (median = 47 Mb), Atrophos_heuristic 19–2,332 Mb (median = 79 Mb), fastp 1,057–2,9240 Mb (median = 1,283 Mb), and EARRINGS (default and sensitive) 6,539 Mb (Fig 4d). The evaluation was performed on a Linux server with 96 cores Intel(R) XeonGold 6246 CPU (3.2GHz) and 1T RAM.

## Availability and future directions

FindAdapt is fast, memory-efficient, and powerful for identifying a variety of adapter patterns, which facilitates small RNA-seq analysis and the reproducibility of publicly available datasets. FindAdapt achieved much better performance than existing approaches since it distinguishes adapters from true sequences by matching reads to a reference instead of identifying over-represented k-mers. FindAdapt is also flexible on adapter patterns by considering random-mers at the 5’ and 3’ ends.

FindAdapt uses miRNA sequences automatically derived from miRBase v22.1 as the reference. Therefore, FindAdapt supports all 271 organisms contained in the miRBase, including human, mouse, rat, fly, and Arabidopsis. We evaluated the performance of FindAdapt on 50 small RNA-seq datasets, which were randomly chosen from the GEO across multiple organisms. As a result, FindAdapt achieved 100% accuracy as well (S2 Table). Besides, FindAdapt provides the option to use a list of sequences as a reference in cases where the miRNA sequences of the organism are not included in the miRBase or when other types of small RNAs rather than miRNAs are the major focus.

FindAdapt achieved 100% accuracy on 3,184 small RNA-seq datasets from human and 50 from non-human organisms. In extreme scenarios when non-template isomiRs become the dominant form, however, FindAdapt might not be able to identify adapters correctly. FindAdapt assumes a general structure of adapters consisting of 0-Nbp random-mer at the 5’, and 0-N bp random-mer at the 3’ ends, followed by adapter sequences (Fig 1f), which covers various adapter patterns from existing small RNA library preparation kits. Further development or adjustment of FindAdapt might be required if a new kit generates an adapter pattern unmatching the assumed general structure.

FindAdapt is freely available at https://github.com/chc-code/FindAdapt. The installation instruction, manual, output format, and examples of FindAdapt are also provided at the GitHub.

## Supporting information

S1 Table Adapter patterns identified by FindAdapt on 87 human small RNA-seq studies. (XLSX)

S2 Table Adapter patterns identified by FindAdapt on 13 studies involving 50 small RNA-seq datasets from non-human species, including mouse, rat, Arabidopsis, rice, and grape. (XLSX)

S1 Text Pseudocode for identifying the adapter sequence and determining 3’ random-mer length. (PDF)
