# Supplementary material for: FindAdapt: A python package for fast and accurate adapter detection in small RNA sequencing
Source: PLoS Comput Biol. 2024 Jan 22;20(1):e1011786. doi: 10.1371/journal.pcbi.1011786 (PMC10833567; doi:10.1371/journal.pcbi.1011786)
Supplement: S1 Text — (PDF) [file pcbi.1011786.s003.pdf]

Algorithm 1: pick_3p_adapter (*top5records*)

Input : top5records : the records with the top 5 highest counts in each shifted position. each record=[seq,shift,count]

Output : the candidate adapter sequence and 3' random-mer length

Parameter: thres_multiplier (default: 1.2)

```
lineage ← { };
for [seq, shift, count] in top5records do
    seq_child ← seq[1:];
    lineage[seq_child] ← [seq, shift, count];
end
candidate ← [ ];
for [seq, shift, count] in top5records do
    if shift == 0 then
        candidate.append([seq, shift, count])
    end
    else
        seq_truncated ← seq[:-1];
        (parent_seq, parent_shift, parent_count) ←
          lineage[seq_truncated];
        if parent_shift < shift and parent_count × thres_multiplier ≥
          count then
            continue;
        end
        else
            candidate.append([seq, shift, count]);
        end
    end
end
candidiate_sorted ← sorted(candidate, key=count, reverse=True);
final_candidate ← candidiate_sorted[0];
return final_candidate;
```
